# 25-hydroxyvitamin D as a predictor of reduced heart rate variability in elderly patients with diabetes mellitus

**DOI:** 10.3389/fendo.2025.1556345

**Published:** 2025-04-30

**Authors:** Tiantian Tang, Xinyu Sun, Xiaoxiang Zhang, Jiaojiao Li, Manjie Wang, Cancan Hui, Yuwei Cheng, Xiaoming Kong, Yan Sun

**Affiliations:** Department of Geriatric Endocrinology, The First Affiliated Hospital of Anhui Medical University, Hefei, Anhui, China

**Keywords:** elderly diabetes, heart rate variability, cardiac autonomic neuropathy, 25-(OH)D, diabetic nephropathy

## Abstract

**Objective:**

To analyze the risk factors for decreased heart rate variability (HRV) in elderly diabetic patients and evaluate the predictive value of 25-hydroxyvitamin D [25-(OH)D].

**Methods:**

A retrospective study was conducted, enrolling 101 elderly diabetic patients admitted to the Department of Geriatric Endocrinology at the First Affiliated Hospital of Anhui Medical University from 2023 to 2024. Patients were divided into two groups based on the standard deviation of all normal RR intervals (SDNN) from 24-hour Holter monitoring: the normal HRV group (180 ms ≥ SDNN ≥ 100 ms) and the decreased HRV group (SDNN < 100 ms). Clinical data collected included age, sex, body mass index (BMI), Diabetic peripheral neuropathy (DPN), Diabetic peripheral vascular disease (DPVD), Diabetic nephropathy (DN), Diabetic retinopathy (DR), as well as laboratory parameters such as fasting plasma glucose (FPG), glycated hemoglobin (HbA1c), creatinine (Cr), glomerular filtration rate (GFR), uric acid (UA), cystatin C (CysC), total cholesterol (TC), triglycerides (TG), high-density lipoprotein cholesterol (HDL-C), low-density lipoprotein cholesterol (LDL-C), very-low-density lipoprotein cholesterol (VLDL-C), non-HDL-C, 25-(OH)D, 24-hour urinary total protein (24hUTP), 24-hour urinary uric acid (24hUA), microalbuminuria (MAlb), urinary albumin-to-creatinine ratio (UACR), and calculated ratios including TG/HDL-C, UA/HDL-C, and non-HDL-C/HDL-C. Statistical analyses included χ² test, independent *t*-test, Mann-Whitney U test, and multivariate logistic regression to identify risk factors for HRV reduction. Receiver operating characteristic (*ROC*) curve analysis was performed to assess the predictive value of these factors.

**Results:**

Compared with the normal HRV group, the decreased HRV group had significantly lower HDL-C and 25-(OH)D levels but higher prevalence of DN, along with elevated 24hUTP, MAlb, UACR, TG/HDL-C, and UA/HDL-C (all P < 0.05). These factors were identified as independent risk factors for HRV reduction in elderly diabetic patients.

**Conclusion:**

This study identified key risk factors for HRV reduction in elderly diabetic patients. Furthermore, 25-(OH)D levels may serve as an early predictive marker for decreased HRV in this population.

## 1 Introduction

With the accelerating global aging population, the prevalence of diabetes in elderly individuals has been increasing annually. This trend not only poses significant threats to the physical and mental health of older patients but also creates a substantial burden on healthcare systems, particularly due to the numerous complications associated with diabetes. Among these, damage to the autonomic nerve fibers innervating the heart and blood vessels, known as diabetic cardiac autonomic neuropathy (DCAN) (1), elevates the risk of adverse cardiovascular events in elderly diabetic patients. However, the early stages of DCAN may be asymptomatic or present with mild symptoms, making it clinically challenging to detect or diagnose during routine medical evaluations. Heart rate variability (HRV) which reflects the combined activity of the sympathetic and parasympathetic nervous systems on cardiac function, is currently recognized as an important non-invasive indicator for assessing cardiac autonomic function (2). HRV is obtained by measuring and analyzing the temporal variations between heartbeats, specifically the R-peak to R-peak intervals (RR intervals) in electrocardiograms (3). The standard deviation of all normal RR intervals (SDNN)serves as an effective parameter for evaluating the status of cardiac autonomic function in diabetic patients (4). Particularly in ambulatory electrocardiographic recordings exceeding 30 seconds, SDNN has been demonstrated to be a reliable metric for assessing DCAN (5).

In recent years, vitamin D has become a research hotspot, and its receptors are widely distributed in various human tissues, including blood vessels, the heart, and the brain (6). Numerous studies have demonstrated that vitamin D deficiency can increase the incidence and mortality of cardiovascular diseases, cerebrovascular diseases, and metabolic disorders (7–9). However, few studies have explored the impact of vitamin D levels on HRV parameters in elderly diabetic patients. Based on this, the present study aims to investigate the risk factors for reduced HRV in elderly diabetic patients and assess its predictive value, with the goal of providing a basis for early intervention and treatment of DCAN.

## 2 Materials and methods

### 2.1 Study design

Participants were stratified according to the HRV criteria recommended by the European Society of Cardiology and the North American Society of Pacing and Electrophysiology, using the standard deviation of SDNN threshold. The cohort comprised 101 elderly diabetic patients, divided into:Normal HRV group (180 ms ≥ SDNN ≥ 100 ms): 59 cases;Reduced HRV group (SDNN < 100 ms): 42 cases (10).

#### 2.1.1 Data collection

The study collected the following clinical data: (1) General information including age, gender, body mass index (BMI), duration of diabetes, and presence of complications such as diabetic peripheral neuropathy(DPN), diabetic peripheral vascular disease(DPVD), diabetic nephropathy(DN), and diabetic retinopathy(DR); (2) Laboratory parameters including fasting plasma glucose (FPG), glycated hemoglobin (HbA1c), serum creatinine (Cr), glomerular filtration rate (GFR), uric acid (UA), cystatin C (CysC), total cholesterol (TC), triglycerides (TG), high-density lipoprotein cholesterol (HDL-C), low-density lipoprotein cholesterol (LDL-C), very low-density lipoprotein cholesterol (VLDL-C), non-high-density lipoprotein cholesterol (non-HDL-C), 25-hydroxyvitamin D [25-(OH)D], 24-hour urinary total protein (24hUTP), 24-hour urinary uric acid (24hUA), microalbuminuria (MAlb), urinary albumin-to-creatinine ratio (UACR), and SDNN. Based on these parameters, the study further calculated the ratios of TG/HDL-C, UA/HDL-C, and non-HDL-C/HDL-C.

#### 2.1.2 Statistical analysis

Data were analyzed using SPSS 25.0.Continuous variables: Normally distributed data expressed as mean ± SD (independent *t*-test); non-normal data as median (P25, P75) (Mann-Whitney U test). Categorical variables: Reported as counts (%) (χ² test). Statistical significance was set at P < 0.05. Multivariate analysis: Logistic regression identified risk factors for HRV reduction. Predictive value: Receiver operating characteristic (*ROC)* curves evaluated 25-(OH)D’s ability to predict HRV decline.

### 2.2 Study subjects

A total of 101 elderly patients with diabetes hospitalized in the Geriatric Endocrinology Department of the First Affiliated Hospital of Anhui Medical University from 2023 to 2024 were enrolled as study subjects. All study participants were selected from indoor workers engaged in light physical labor, with potential confounding factors such as physical activity, sunlight exposure, and dietary habits already controlled for. During both the pre-monitoring period and monitoring period, participants were required to: Avoid emotional excitement and vigorous physical activities; Abstain from consuming any caffeine-containing foods or beverages.

### 2.3 Inclusion and exclusion criteria

The study subjects were elderly diabetic patients hospitalized in the Department of Geriatric Endocrinology at the First Affiliated Hospital of Anhui Medical University from 2023 to 2024. Inclusion criteria: a. Aged 60 years or older; b. Meeting the WHO diagnostic criteria for diabetes and clinically confirmed as diabetic patients; c. Able to cooperate with dynamic electrocardiogram monitoring. Exclusion criteria:a. Patients with secondary diabetes caused by conditions such as Cushing’s syndrome, hyperthyroidism, or pancreatic diseases; b. Those with comorbid cognitive impairment; c. Those with severe cardiovascular diseases, such as acute or chronic heart failure, cardiomyopathy, acute coronary syndrome, rheumatic heart disease, congenital heart disease, or valvular heart disease; d. Those with severe respiratory diseases, severe infections, trauma, stress, hepatic or renal dysfunction, or severe neuropsychiatric disorders; e. Those who received intravenous glucose or glucocorticoids (or other glucose-affecting medications) within one month before admission or during hospitalization; f. Those with diseases affecting the autonomic nervous system, such as acute inflammatory demyelinating polyneuropathy or Parkinson’s disease; g. Those who recently took medications affecting HRV, such as α- or β-blockers or statins; h. Those unable to cooperate with dynamic electrocardiogram monitoring due to physical or mental reasons.

## 3 Results

### 3.1 Comparison of baseline data and laboratory indicators between groups

Compared to the HRV-normal group, the HRV-reduced group demonstrated significantly lower levels of HDL-C and 25-(OH)D, along with a higher number of diabetic nephropathy cases, elevated 24hUTP, MAlb, UACR, TG/HDL-C, and UA/HDL-C ratios (all P < 0.05; see **Table 1**).

**Table 1:** Comparison of baseline characteristics and laboratory parameters between groups.

Variable	HRV-normal group (n=59)	HRV-reduced group (n=42)	Test Statistic	P-value
Age/year	70 (66,75)	67.5 (61,78.25)	Z=-0.559	0.576
Sex [n (%)]			χ2 = 0.09	0.764
Man	41 (59.4%)	28 (40.6%)		
Woman	59 (56.2%)	14 (43.8%)		
DPN	40 (59.70%)	27 (40.3%)	χ2 = 0.135	0.713
DPVD	54 (59.30%)	37 (40.7%)	χ2 = 0.053	0.817
DN	15 (41.7%)	21 (58.3)	χ2 = 6.46	0.011
DR	4 (100%)	0 (0%)	χ2 = 1.45	0.228
BMI/ (kg·m^−2^)	23.91 (21.91,25.46)	24.09 (22.18,25.95)	Z=-0.158	0.875
Disease duration	13 (8,21)	14.5 (4.5,23.25)	Z=-0.297	0.767
FPG	7.44 (5.995,8.53)	7.1 (5.47,8.36)	Z=-0.604	0.546
HbA1c	7.55 (6.7,8.38)	7.55 (6.5,8.93)	Z=-0.012	0.991
Cre	73 (63,94)	73 (61.75,89.5)	Z=-0.034	0.973
GFR	83 (73.75,99.25)	91 (70.75,100.25)	Z=-0.213	0.831
UA	331 (279.25,378.5)	323 (286,391.5)	Z=-0.101	0.919
CysC	0.88 (0.755,1.06)	0.9 (0.73,1.15)	Z=-0.632	0.527
TC	3.93 (3.18,5.26)	3.81 (3.18,4.36)	Z=-0.985	0.325
TG	1.31 (0.88,1.65)	1.43 (1,2.33)	Z=-1.475	0.14
HDL-C	1.13 (0.96,1.35)	1 (0.85,1.13)	Z=-2.856	0.004
LDL-C	2.21 (1.72,3.38)	2.09 (1.54,2.56)	Z=-1.412	0.158
VLDL-C	0.48 (0.33,0.61)	0.53 (0.37,0.86)	Z=-1.507	0.132
non-HDL-C	2.65 (2.15,3.69)	2.71 (2.2,3.37)	Z=0	1.00
25- (OH)D	22.24 ± 7.1	16.24 ± 6.65	*t*=3.886	0.00
24hUTP	0.11 (0.08,0.15)	0.17 (0.11,0.34)	Z=-2.671	0.008
24hUA	2887.37 ± 1033.53	2604.88 ± 1507.51	*t*=0.984	0.329
MAlb	5.87 (1.36,19.13)	16.98 (7,75.54)	Z=-3.29	0.001
UACR	0.87 (0.23,1.88)	2.68 (1.21,16.01)	Z=-4.148	0.00
UA/HDL-C	266.51 (219.22,352.71)	332.14 (248.69,453.22)	Z=-2.305	0.021
TG/HDL-C	0.96 (0.78,1.77)	1.26 (0.98,2.7)	Z=-2.257	0.024
non-HDL-C/HDL-C	2.44 (1.74,3.15)	2.56 (2.05,3.82)	Z=-1.332	0.183

DPN, Diabetic peripheral neuropathy; DPVD, Diabetic peripheral vascular disease; DN, Diabetic nephropathyl; DR, Diabetic retinopathy; FPG, fasting plasma glucose; HbA1c, glycated hemoglobin; Cr, creatinine; GFR, glomerular filtration rate; UA, uric acid; CysC, cystatin C; TC, total cholesterol; TG, triglycerides; HDL-C, high-density lipoprotein cholesterol; LDL-C, low-density lipoprotein cholesterol; VLDL-C, very low-density lipoprotein cholesterol; non-HDL-C, non-HDL cholesterol; 25- (OH)D, 25-hydroxyvitamin D; 24hUTP, 24-hour urinary total protein; 24hUA, 24-hour urinary uric acid; MAlb, microalbuminuria; UACR, urinary albumin-to-creatinine ratio; TG/HDL-C, triglyceride-to-HDL-C ratio; UA/HDL-C, uric acid-to-HDL-C ratio; non-HDL-C/HDL-C, non-HDL-C-to-HDL-C ratio.

### 3.2 Multivariate logistic regression analysis

The model showed good goodness-of-fit with Nagelkerke R² of 0.343 and Cox & Snell R² of 0.255. All tolerance values were >0.1 and variance inflation factors (VIF) were <5, indicating no multicollinearity among independent variables. Furthermore, the model met all necessary assumptions: (a) the dependent variable was binary, (b) there was linearity between independent variables and Logit transformation, (c) observations were independent, (d) sample size was adequate, (e) no extreme outliers were present in the data, and (f) no important variables were omitted from the model, thus validating the use of logistic regression analysis.

Using HRV status as the dependent variable (normal=0, abnormal=1) with DN cases, HDL-C, 25-(OH)D, 24hUTP, MAlb, UACR, TG/HDL-C, and UA/HDL-C as independent variables, logistic regression analysis showed only 25-(OH)D had P<0.05, indicating statistically significant association. 25-(OH)D was a protective factor against HRV decline (odds ratio [OR]=0.869, 95% confidence interval [95%CI] 0.778-0.97). Other variables (DN, HDL-C, 24hUTP, MAlb, UACR, TG/HDL-C, UA/HDL-C) all showed P>0.05, indicating no statistical significance in this model (see **Table 2**).

**Table 2:** Logistic regression analysis of HRV reduction.

Dependent variable	β-value	S.E.	Wald-value	OR-value	95%CI	P-value
DN	0.724	0.73	0.983	0.485	0.116∼2.028	0.321
HDL-C	-1.499	1.528	0.962	0.223	0.011∼4.463	0.327
25-(OH)D	-0.141	0.056	6.284	0.869	0.778∼0.97	0.012
24hUTP	-0.201	0.315	0.406	0.818	0.441∼1.518	0.524
MAlb	-0.009	0.008	1.39	0.991	0.976∼1.006	0.238
UACR	0.025	0.024	1.085	1.025	0.978∼1.074	0.298
TG/HDL-C	0.061	0.306	0.04	1.063	0.584∼1.936	0.842
UA/HDL-C	-0.001	0.003	0.032	0.999	0.993∼1.006	0.858

DN, Diabetic nephropathy; HDL-C, high-density lipoprotein cholesterol; 25-(OH)D, 25-hydroxyvitamin D; 24hUTP, 24-hour urinary protein; MAlb, urinary microalbumin; UACR, urinary albumin-to-creatinine ratio; TG/HDL-C, triglyceride-to-HDL-C ratio; UA/HDL-C, uric acid-to-HDL-C ratio.

### 3.3 *ROC* curve analysis

**Figure 1:**
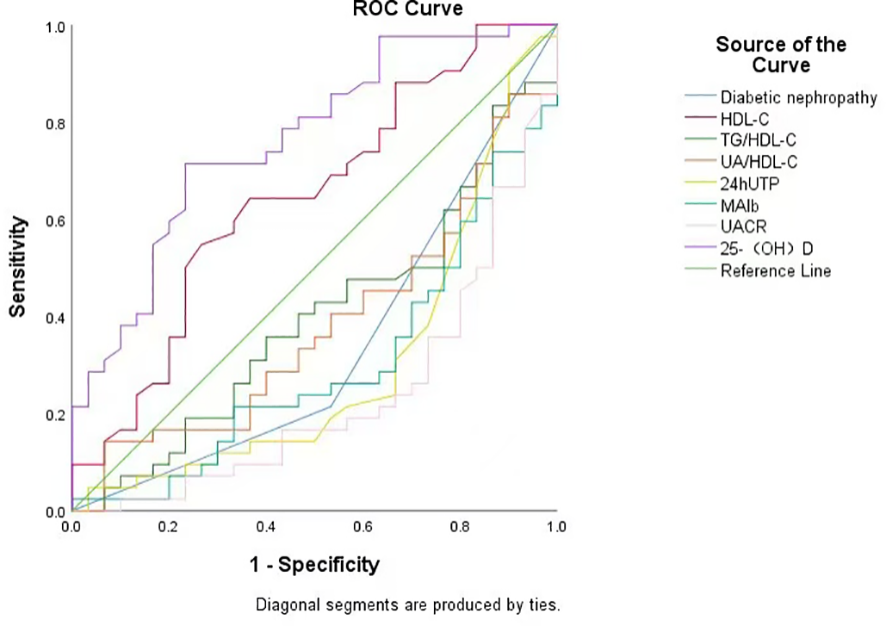
*ROC* curves of DN status, HDL-C, 24hUTP, MAlb, UACR, TG/HDL-C, and UA/HDL-C levels for diagnosing and predicting HRV decline in elderly patients with diabetes mellitus.

To evaluate the predictive value of DN, HDL-C, 24hUTP, MAlb, UACR, TG/HDL-C, and UA/HDL-C for HRV decline in elderly diabetic patients, we performed *ROC* curve analysis. The results showed that 25-(OH)D had the highest predictive efficacy (area under the curve [AUC]=0.763, P=0.00, 95% CI 0.653-0.874), with a sensitivity of 67.3%, specificity of 76.5%, and maximum Youden index of 43.8%. The optimal predictive cutoff value for serum 25-(OH)D determined by the maximum Youden index was 19.7 ng/mL (reference normal range >20 ng/mL), with positive predictive value [PPV] of 61.9% and negative predictive value [NPV] of 80.5%. Therefore, 25-(OH)D levels have certain predictive value for diagnosing HRV decline in elderly diabetic patients (see **Figure 1**, **Table 3**).

**Table 3:** Area under the curve.

Test Result Variable(s)	Area	P-value	95%CI
DN	0.34	0.022	0.21∼0.471
HDL-C	0.644	0.039	0.513∼0.774
25-(OH)D	0.763	0	0.653∼0.874
24hUTP	0.301	0.004	0.172∼0.43
MAlb	0.3	0.004	0.178∼0.422
UACR	0.235	0	0.122∼0.347
TG/HDL-C	0.396	0.136	0.265∼0.528
UA/HDL-C	0.382	0.089	0.251∼0.512

DN, Diabetic nephropathy; HDL-C, high-density lipoprotein cholesterol; 25-(OH)D, 25-hydroxyvitamin D; 24hUTP, 24-hour urinary protein; MAlb, urinary microalbumin; UACR, urinary albumin-to-creatinine ratio; TG/HDL-C, triglyceride-to-HDL-C ratio; UA/HDL-C, uric acid-to-HDL-C ratio.

## 4 Discussion

Currently, DCAN diagnosis primarily utilizes the Ewing test, which consists of five non-invasive assessments: deep breathing heart rate [HR] variation, supine-to-standing HR change, orthostatic blood pressure [BP] difference, Valsalva maneuver, and handgrip test (11). However, this approach has notable limitations including procedural complexity, limited availability, and substantial subjectivity. In comparison, HRV analysis offers a more sensitive and non-invasive evaluation of cardiac autonomic function, providing objective, convenient, and reproducible results through 24-hour Holter monitoring. Multiple studies have confirmed that early HRV monitoring facilitates timely detection and diagnosis of cardiovascular autonomic neuropathy (2, 12). Consequently, proactive intervention targeting HRV decline risk factors, when combined with comprehensive management of diabetic cardiovascular risks, may potentially delay or prevent DCAN progression (13). Our retrospective study identified DN comorbidity, HDL-C, 24hUTP, MAlb, UACR, TG/HDL-C, and UA/HDL-C as significant risk factors for HRV decline in elderly diabetic patients, while 25-(OH)D levels demonstrated predictive value for early identification of HRV impairment in this population.

Multiple prospective clinical and epidemiological studies have confirmed a negative correlation between HDL-C and the occurrence and severity of atherosclerosis (14, 15). Additionally, HDL-C plays a role in maintaining normal vascular function (16). Decreased HDL-C may exacerbate arterial stiffness and damaging cardiac microvessels, while the occurrence of reduced HRV in diabetic patients may be related to cardiac microvascular lesions (17). Therefore, higher HDL-C levels can reduce the risk of HRV decline. Studies have shown that a high TG/HDL ratio is closely associated with insulin resistance and atherosclerosis in prediabetic and newly diagnosed diabetic subjects (18, 19). Thus, elevated TG/HDL-C levels may contribute to cardiac microvascular damage and exacerbate diabetes-induced autonomic nerve dysfunction, leading to reduced HRV. UA, the end product of purine metabolism, can influence the progression of cardiac autonomic neuropathy (20). Since UA/HDL-C combines both the damaging and protective mechanisms of cardiac autonomic nerves, UA/HDL-C levels are closely related to HRV decline. Our study found that the HRV decline group had significantly lower HDL-C and significantly higher TG/HDL-C and UA/HDL-C levels, suggesting that lipid and purine metabolism disorders in elderly diabetic patients are correlated with DCAN. Therefore, managing lipid and UA levels should be emphasized throughout diabetes care.

DN is a common complication of diabetes. Studies indicate that DN markers including urinary albumin excretion and UACR impairment coexist with autonomic neuropathy and reduced HRV (21). The development of DN may be associated with underlying inflammatory effects of microvascular complications and cardiorenal syndrome (22). Given the relatively consistent progression of systemic microvascular damage in diabetic patients, we hypothesize that the presence of DN, elevated UACR and urinary albumin levels may reflect the progression of cardiac microvascular dysfunction. Our results show significantly higher DN incidence, 24hUTP, MAlb and UACR levels in the reduced HRV group, suggesting that DCAN may develop concurrently with DN.

Recent studies have shown that vitamin D plays a significant role in various cardiovascular diseases, including Coronary artery disease (CAD) and heart failure (HF) (23). However, few studies have identified the precise threshold of 25-(OH)D for predicting HRV reduction. Our results indicate that 25-(OH)D is a protective factor against HRV decline (OR=0.869). A serum 25(OH)D level of 19.7 ng/mL demonstrated a PPV of 61.9% and an NPV of 80.5% for predicting HRV decline in elderly diabetic patients. The study by Xuemei Luo et al. suggested that vitamin D deficiency leading to reduced HRV may be associated with the following mechanisms (24):a. Vitamin D can regulate cardiovascular activity by coordinating other molecular mechanisms (e.g., neurotransmitter biosynthesis), as it controls the synthesis of acetylcholine (ACh) and the expression of tyrosine hydroxylase (TH).Vitamin D-deficient rats exhibited decreased TH in cardiac regions and reduced ACh in local cardiac plasma, along with corresponding declines in Nitric oxide (NO) and norepinephrine (NE). ACh slows heart rate, delays atrioventricular node conduction, weakens atrial muscle contraction, and promotes NO release and vasodilation. NE is the primary neurotransmitter of sympathetic nerves, while TH is a rate-limiting enzyme in NE and epinephrine biosynthesis.b. Vitamin D deficiency may reduce potassium channel protein levels by downregulating the expression of inwardly rectifying potassium channel (Kir), human ether-à-go-go-related gene potassium channel (HERG), K^+^ voltage-gated channel subfamily Q member 1 (KVLQT1), and minimal potassium channel subunit (MinK), thereby disrupting cardiac autonomic homeostasis. Furthermore, decreased 25(OH)D levels may lead to reduced HRV through the following mechanisms:a. As the primary active form of 25(OH)D, reduced calcitriol levels may promote inflammatory factor release in the paraventricular nucleus (PVN) and increase systemic oxidative stress, thereby inducing autonomic dysfunction (25, 26).b. 25(OH)D serves as an inhibitor of renin biosynthesis. Its deficiency can increase production of both renin and angiotensin II (Ang II) (27). Moreover, elevated serum parathyroid hormone (PTH) levels secondary to 25(OH)D deficiency may further stimulate renin secretion (28). The combined effects of 25(OH)D deficiency and increased PTH may activate the renin-angiotensin-aldosterone system (RAAS), resulting in sympathetic and parasympathetic dysfunction that ultimately affects HR. Currently, few studies have demonstrated that autonomic dysfunction can influence 25(OH)D metabolism. However, the report by Nalbant et al. (29) indicated no significant alterations in HRV parameters among vitamin D-deficient individuals within the low cardiovascular risk group. This finding contradicts the results of the present study. The discrepancy may be attributed to the following factors: the mean age of participants in prior studies was younger than that in our investigation, and previous study cohorts exhibited lower cardiovascular risk profiles, whereas our research specifically focused on elderly patients.

Currently, there is no effective reversal strategy for elderly diabetic patients with declined HRV, making early prevention, diagnosis, and intervention crucial. Studies have shown that vitamin D supplementation significantly improves cardiac autonomic dysfunction and glucose tolerance in vitamin D-deficient individuals (30, 31). Given its affordability and convenience, vitamin D supplementation for elderly diabetic patients with low 25-(OH)D levels may be a potential therapeutic approach to improve HRV in this population.

This study has several limitations: (a) relatively small sample size; (b) as a cross-sectional design, longitudinal data on 25-(OH)D levels and HRV decline in elderly diabetics are unavailable. Future studies should incorporate larger cohorts with extended follow-up to monitor 25-(OH)D/HRV dynamics, coupled with vitamin D supplementation trials, to better establish 25-(OH)D’s predictive value for HRV impairment and evaluate its therapeutic potential for early-stage DCAN intervention.

In summary, elderly diabetic patients with reduced HRV showed lower serum 25-(OH)D levels than those with normal HRV, suggesting 25-(OH)D may serve as a potential biomarker for HRV impairment. DN, HDL-C, 25-(OH)D, 24hUTP, MAlb, UACR, TG/HDL-C, and UA/HDL-C were all significantly associated with HRV reduction in this population.

## Data Availability

The original contributions presented in the study are included in the article/supplementary material. Further inquiries can be directed to the corresponding authors.
